# Why Delusional Infestation should remain Delusional Infestation

**DOI:** 10.4269/ajtmh.19-0927

**Published:** 2020-03

**Authors:** Peter Lepping, Stephen L. Walker, Anthony P. Bewley

**Affiliations:** Wrexham Maelor Hospital Psychiatric Liaison Team; Betsi Cadwaladr University Health Board; Heddfan Psychiatric Unit; Wrexham, United Kingdom; Centre for Mental Health and Society; Bangor University; Bangor, United Kingdom; Mysore Medical College and Research Institute; Mysore, India; E-mail: peter.lepping@wales.nhs.uk; Hospital for Tropical Diseases and Department of Dermatology; University College London Hospitals NHS Foundation Trust; London, United Kingdom; Department of Clinical Research; London School of Hygiene and Tropical Medicine; London, United Kingdom; E-mail: steve.walker@lshtm.ac.uk; Department of Dermatology; Barts Health NHS Trust; London, United Kingdom; Queen Mary University of London; London, United Kingdom; E-mail: anthony.bewley@nhs.net

Dear Sir,

We read with interest the editorial by Matthew Grant about delusional infestation (DI).^1^ We have been involved in the treatment of DI for some years. We lead specialist clinics^2,3^ and have been involved in the recent development of national guidelines through the British Association of Dermatologists (BAD). We agree with Grant’s summary of the difficulties that arise when treating patients with delusional beliefs. We also agree that significant risks can arise in patients with DI, both to themselves and to others through attempts to get rid of the alleged pathogens. However, we do not agree with the conclusion that the name DI should be changed, and it was interesting to note that the editorial did not suggest any alternatives. The complexity of the treatment challenges are somewhat missed in the editorial. Two of us have been party to advocating a change in the nomenclature to DI from previous names because they did not encompass varying changes in alleged pathogens.^4^ Our reasons are as follows:

First, various changes to the terminology have been attempted without improving the inherent difficulties that arise when treating patients with mono-delusional beliefs. Delusional infestation is a delusional disorder, and a change of terminology will not alter this. It is important for research and practical purposes to be as accurate as possible with a description of the disease.

Second, most experts agree that clinicians should use symptom description for DI and similar illnesses at the starting point of discussions with patients and, thus, initially avoid the term delusional.^5^ The new BAD national guidelines for DI incorporate this approach. As patients usually lack capacity to make treatment decisions because of their intense delusional belief, it is ethically justified to have a gradual approach to diagnosis disclosure. This is usually in the patient’s best interest and common practice with similar illnesses with high levels of disease burden and lack of insight. Sensitivity is needed to create engagement and rapport with the patient, which facilitates effective treatment, that is, antipsychotic medication.

We do not agree that a change to the terminology would be helpful or improve patient engagement. A Cochrane systematic review of the treatment of primary DI^6^ is available, and the BAD guidelines are imminent. These guidelines are evidence based, to the degree that evidence currently exists. In specialist clinics and other settings, where these guidelines have been adhered to, outcomes have been promising and very much better than outcomes in standard settings.

We agree with Grant that psychiatric referrals alone are usually ineffective. We have recently shown that a longer duration of untreated illness is associated with worse outcomes.^7^ All practitioners who see affected individuals should focus on early intervention that requires early engagement and rapport with the patient to facilitate meaningful and effective treatment.^8^ We support any attempts to improve this challenging condition.
